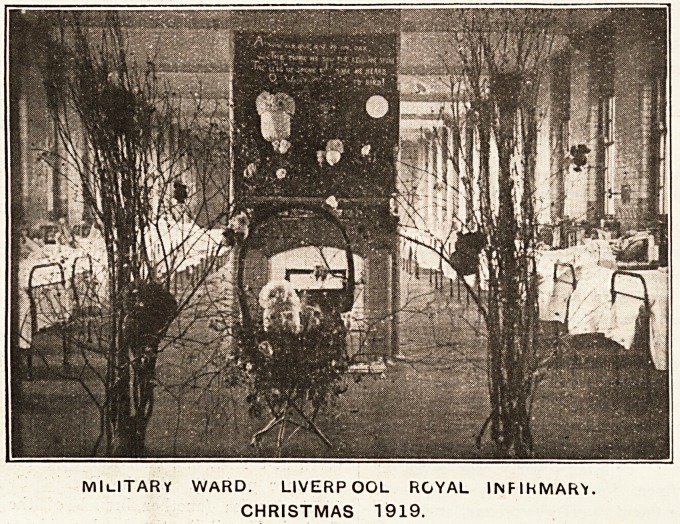# Christmas in the Hospitals

**Published:** 1920-01-03

**Authors:** 


### CHRISTMAS IN THE HOSPITALS

### GUY'S HOSPITAL IN CHRISTMAS GARB.

### Fields of Beauty, Artistic Design, and Unfailing Mirth.

No hospital worker in Great Britain, to say nothing of the outer world, can realise what it really means to be in a happily joyous and contented hospital, what artistic gifts lie buried beneath the caps and aprons of hospital sisters and nurses, or the power Christmas gives to hospital patients whatever their ailments, who have not had the privilege of seeing hospital wards in Father Christmas's possession and control, as they, in fact, are to be seen at Guy's Hospital, in St. Thomas's Street, London. In our preliminary inquiries we were told that it was not anticipated that it would be possible to make much of Christmas in the borough of Southwark this year. The war had left its mark upon everything and everybody, the people available for enlistment under Father Christmas's command were fewer and less keen than the men from the Front; the weather promised to be very uninviting; the number of patients was so great as to overflow the beds; and human zeal and enterprise could not flourish under such conditions, much less produce beautiful effects and make creations which would include the wild beasts of the forest, the animals of the veldt, black and yellow dragons with mandarins in attendance, and other celestial beings of Chinese origin. Hope, however, reigns eternal in the human breast, and keen resolve to let no difficulty impede the joyous progress of Father Christmas within the walls of Guy's Hospital overcame every obstacle, and produced many triumphs in ward decoration, to say nothing of new features in the way of clothing presented by numerous members of the troupe of Guy's Minstrels, who made the wards merry from 11 a.m. to 7 p.m. on Christmas Day.

We have not space to give word-pictures of the wards as we should wish to do, but it is our good fortune to have a brief description of the decorations in the whole of the wards from the pen of a very able writer, who is deservedly one of the most popular of high officials, and was whole-heartedly engaged with Father Christmas in making things hum everywhere throughout the Hospital. We have ventured to place in special type descriptions of seven of the wards where originality, the mastery of colour, the triumph of simple decoration, the successful surmounting of impossible conditions in the creation of an old-world garden, some of Nature's designs, the conversion of sunshades into lampholders, the creation of a home for Chinese dragons in black, yellow dragons, and other celestial beings in, of all places, the eye ward. Orange bowl lampshades was a bold adventure; illustrated nursery rhymes, and lampshades of illuminated houses ran Queen's hard, but then the ambitious competitor was no less than Victoria, who had under her wing the old woman who lived in a shoe, with that mischievous imp little Pollie Flinders, and the adventuresome and sturdy Jack and the Beanstalk. Yes, there was much to see, to study, to learn, to enjoy, to wonder at, rejoice over, and amidst it all to become alive to Christmas possibilities and the splendid outpouring of Personal Service, Good Fellowship, Heartfelt Sympathy for the Sick, and all that is best in human nature, to be found, and thank God for, in a tour of Guy's Hospital by the observant on Christmas Day, 1919.

The writer of this brief note was brought up sharply at the outset of this effort to place on paper some idea of Guy's Hospital in Christmas garb. The second line as dictated read:—Fields of Beauty, Artistic Designs and *Unfailing Mirth*. As transscribed it presented the reader with *Unveiling Mirth*, a costume which we could not properly describe in detail in the columns of The Hospital, much less present pictorially in *propria persona*.

We now proceed to give the synopsis of the decorations in each ward, for which we have already expressed our indebtedness to the splendid worker who, we have it from Father Christmas, did so much to encourage and cheer every patient, not forgetting the 700 children in Out-Patients' during the merry time, wonderful scenes and singing, on the afternoon of Christmas Day.

### WARD DECORATIONS

### Surgical Block.

Astley Cooper.—Light festoons of ivy. Lights shaded with bunches of grass, yellow-centre, cornflowers, and poppies. Small lights concealed in beds of cornflowers and poppies on the tables.

Cornelius.—Pear-shaped orange lampshades. Light festoons of red and orange autumn leaves, holly, and ivy, a large Christmas-tree.

Lydia.—Festoons of ivy and holly. Large pale-pink circular lampshades. Small pink hand-lamps.

**Job.—Square, old-fashioned yellow lampshades embroidered with black witches. Holly and ivy decorations. Very bright and simple.**

Lazarus.—Lampshades of orange waterlilies and orange lanterns. Tubs of hollyhocks and evening primroses.

Martha.—Square, cream lampshades to resemble leaded panes. Tudor-rose conventional design. Pink-paper rambler-rose festoons, holly and ivy.

Charity.—Large, open rose-petal lampshades. Festoons of red and rose flowers, holly and ivy.

**Luke.—Old lattice-work rose and black lampshades. Ward transformed into an old garden. Dovecote, beehive, water garden, sundial, rambler-rose arches, birds, stone-bath, and rose-beds.**

**Naaman.—Lampshades made of small rose-coloured sunshades. Light festoons of Ivy, tubs of many-coloured azaleas. The most original decoration in the hospital.**

Dorcas.—Large pink-petal lampshades. Rambler-rose festoons. Small concealed lights in masses of roses on the tables.

Evelyn.—Holly and ivy. Many foreign birds perched about. Lampshades of orange decorated with birds.

Patience.—Holly and ivy festoons, and pots of flowers hiding small lights. Large, square lampshades of orange, banded with brown.

**Christmas in the Hospitals—(*continued*).**

### Medical Block.

EYE WARDS.—**Large, square, yellow lampshades decorated with Chinese designs in black; black and yellow dragons, mandarins, and other celestial beings scattered about the ward.**

MARY.—Violet triangular lampshades. Butterfly designs on screens and walls, holly and ivy.

ADDISON.—Large, red lampshades. Frieze of black imps of different shapes and characters round the central hall. Dragon designs, holly and ivy. Concealed lights.

ESTHER.—Orange lampshades decorated with witches and black cats. Festoons of holly and ivy.

STEPHEN.—Yellow-daffodil lampshades. Festoons of ivy.

BRIGHT.—**Orange-bowl lampshades suspended by orange ribbons. The yellow walls of ward decorated with bare branches and twigs, and a few deep-orange and gold beech leaves. Very simple and effective.**

QUEEN VICTORIA.—QUEEN—**An ostrich farm. Lampshades of straw. Models of farmhouses on the veldt, native labour, and a flock of ostriches—all made by the nurses. Festoons of feathers.**

VICTORIA.—**Illustrated nursery rhymes. Lampshades of illuminated Houses that Jack Built; models of the Three Bears eating porridge; the Old Woman who lived in a Shoe; Little Pollie Flinders; Jack and the Beanstalk; and many others.**

MIRIAM.—Festoons of holly and ivy and green imps. A large Christmas-tree.

ISOLATION.—Dutch lampshades and a frieze of Dutch girls and boats. Blue and white designs. Festoons of holly and ivy, and a Christmas tree. Carols by Southwark Cathedral Choirboys (6.30 to 9 P.M.). The choirboys from Southwark Cathedral came round to the hospital on December 23 singing carols from 6.30 to 9 P.M. They gave great pleasure to the patients and hospital staff.

---

### THE LONDON HOSPITAL.

### One of the Best Christmas Days Known.

BY THE SECRETARY.

PROBABLY for the majority of grown-up people Christmas Day is tinged with sadness—it brings a rush of memories which engender what is colloquially known as the "hump." I would recommend people suffering from this complaint to spend their Christmas Days at the London Hospital—there they will find an atmosphere of happiness where gloomy introspection finds no place. There everyone's energies are absorbed in giving the patients the happiest time of their lives. The "London" has its own time-honoured traditions about celebrating Christmas festivities; the war unfortunately played havoc with these traditions, but they were too strong to be utterly extinguished, and this year they reappeared with the vigour of pre-war times. Here is the programme in brief: 4 A.M., carol-singing round the wards; 9 A.M., Father Christmas visits the wards; noon, Christmas dinner; 3.30 P.M., entertainments begin; 7 P.M., last entertainment concludes.

There are other festivities, such as a supper for night scrubbers at midnight on Christmas Eve, and a dance for the Nurses' Home maids in the out-patient hall, but these are side-shows. The carols—the old carols one has known since childhood—are sung by a choir of sisters and nurses, who pass singing through all the wards of the hospital—a long journey, for there are over sixty wards. Bad luck on the patients to be wakened at 4 A.M., a cynic will exclaim, but ask the patients themselves, and you will find that they would not have had it otherwise for worlds.

At 9 A.M. the appearance of Father Christmas, impersonated by the senior resident medical officer, is hailed with acclamation. His attendant clowns push round a huge trolley which contains the Christmas present for each patient, and this means between 900 and 1,000 packages—for grown-ups some useful garment, and for the children, poor East-enders most of them, toys such as they have only possessed in dreams. At noon, the Christmas dinner. This caused the authorities some anxiety, for there was a danger of the traditional turkey going by the board, owing to prohibitive cost. Friends, however, came to the rescue, and agreed to defray the difference between the cost of turkeys and the ordinary beef diet. A bill of £100 was the result, but the tradition was saved, and turkey and plum pudding was the fare. The resident doctors are called upon to demonstrate the anatomy of the turkey. Each, provided by the sister with a chef's cap and apron, acts as carver in his particular ward.

Then at 3.30 the entertainments begin, and again the resident doctors are the chief performers. It is no easy matter to arrange for all patients to be grouped for the performances, yet the difficulty is overcome. On each floor of the vast wings of the hospital, at the furthermost end of the ward, there is a stage with footlights, and round the stage are grouped all the patients of that floor. A strange sight it is from the stage, that of an audience mostly in bed in "Fowlers." There are fourteen such stages, and fourteen pianos have to be hired from somewhere—no easy matter in these days. On each stage there is a different performance every half-hour, and the total performances in the hospital from 3.30 to 7 were no less than 100.

Chief among the troupes is the resident band. From time immemorial all the house doctors at Christmas have formed themselves into a band with strange and wonderful instruments which they buzz down. The band is generally designated by some title, the significance of which is hid from the lay mind; this year it was called the "Hæmorrhagics." Their costume varies according to what Simmons has in stock. This year they were attired in hunting garb, and all looked very smart in pink; nor was the disguise so complete that a patient failed to recognise "his" doctor. A sketch of the "London" fifty years hence, when all the staff were ladies, and the sisters and nurses men, provided excellent fooling and roars of laughter, and then there was the song with topical verses, with gentle digs at the foibles of the physicians and surgeons.

Besides the "Hæmorrhagics," there were the "Spectra" and the "Scarlet Runners" from the College, and also "Alice in the Wards," a troupe of lady students, the first of its kind at the "London," who were tremendously popular. There were also helpers from outside, violinists, singers, and others, who worked no less enthusiastically than those whose job is at the hospital or college. Last of all there was Punch and Judy for the children's wards, without which no Christmas would be complete. By 7 P.M. the last entertainment had concluded; by 8 P.M. the beds were rearranged, and the wards perfectly quiet and as normal, but for the decorations. Tired but happy, sisters and nurses were talking over one of the best Christmas Days the "London" had ever known.

In the midst of all these festivities the work of the hos-

**Christmas in the Hospitals**—(*continued*).

pital goes on; there is the quiet ward where the "full duty" cases are sent, and in a receiving-room garlanded with evergreen you will find the doctor on duty with sister and nurses attending to the latest accident. A strenuous time is Christmas for doctors, sisters, and nurses, but then the "London" demands hard work at all and every season.

None of the expenses of Christmas come from the general funds. They are defrayed by special contributions. H.M. the King, with his usual thoughtfulness, sends a subscription, and a docker who was in "Rowsell" years ago still sends half-a-crown.

It is a great day of reunion. The indicator in the front hall shows that most of the staff have left the West End and are spending Christmas Day at the hospital; past sisters and nurses, "Londoners" in practice, come to visit the old hospital, so lasting and irresistible is the affection the "London" has on its workers.

### ST. BARTHOLOMEW'S HOSPITAL.

Christmas comes but once a year—in hospitals as in other places—and there, as elsewhere, is rendered memorable by more than the mere infrequency of its occurrence. This last, our first real peacetime Christmas, proved exceptionally successful. By the evening of the 24th students and nursing staff had completed the decorations of the wards. Holly, ivy, fir-needles, and roses draped the curtain rails over the beds, the door lintels, and the passage ways. One very effective scheme comprised artificial birds, of pretty plumage, whose wings moved in a most realistic way. Fairy lights, lanterns, and fancy scrolls made the whole building a riot of tasteful, though exuberant, colour. Medical Out-patients' Hall was unrecognisable to its recent *habitués*; as armchairs, small tables, and a veritable quiltwork of carpets made of it a most effective reception-room for the numerous guests on the Great Day. Pianos were in evidence in all the wards, and called forth unsuspected skill. Mistletoe was to be seen by the careful observer, whilst the very lifts were like fancy illuminated rockets.

On Christmas Day itself Santa Claus, life size, went round the institution on a life-size hobby-horse; cheering and heartening many more than the actual little recipients of his favours. It is a sad fact—but true—that the dinner then provided resulted in many of those "Boxing-Day stomachs" by no means unknown elsewhere. All patients were then moved to the front wards, and the excitement was such that the whole hospital could not boast of a single person who looked *really* ill. Nursing staff and inmates were ready, in advance, to make an unqualified success of the real business of the day.

At 2 p.m. the five troupes of entertainers went round. With the exception of one, the "Roland Ramblers"—which visited the hospital all through the war—all were organised and mainly manned by students. "The Optimists," a turn arranged by the Editor of the hospital *Journal*, gave an exceptionally fine show. Their final operetta—by Signor Acro Megalo—wherein they revived their two corpses with castor oil to the time-honoured refrain "Bart's patients never die . . . they simply fade away" was alike appreciated by students, staff, and patients. The five turns, between them, visited all the forty wards—an arduous task—giving in each good comic turns, dancing, serious and comic, and some very good straightforward singing, remarkably well accompanied. None of the turns would have disgraced a London stage, and no place could have shown a more keenly appreciative audience. One of the most difficult duties the entertainers had to face was to thankfully receive the congratulations which were showered upon them—and to dispose of the tea which every sister insisted on dispensing. Many of the senior staff "looked in"—and stopped longer than they meant, and those students who brought friends and relatives surprised them by the excellence of the programmes and the enthusiastic appreciation of the audiences. The delight of the children would, of itself, have repaid the performers' efforts, and their joy over the Christmas-trees provided was so exuberant as to be almost pathetic.

Everyone voted the show a tremendous success. Certainly as an example of what the staff and students can do when the occasion demands it. It astonished and gratified the patients, and lent fresh enthusiasm to all connected with the hospital; giving them a worthy example and incentive for the efforts of the coming New Year. Thomas Adam (Students' Union).

### KING'S COLLEGE HOSPITAL.

On the three days preceding Christmas carols were sung in the corridors near all the wards. On December 25, after the patients had received their "Post" and most of them their presents, in each and every ward the physicians and surgeons appeared garbed in wonderful, and in some cases fearful, costumes; then on the stroke of noon the signal for "General advance" was given: the great attack on "Turkey" was begun: all the objectives were speedily obtained, without casualties. This great attack was followed by a further advance by sisters and nurses in close column formation, bringing up the plum-pudding and dessert in great variety. A little later the whole hospital resounded with the firing of crackers Each patient was allowed to have two visitors between 3 and 5 p.m., when they were all entertained at tea. When bed-time arrived, the children no less than the adults agreed that, notwithstanding their forced absence from the family circle at their own home, King's College Hospital had given them, indeed, a glorious time.

On Boxing Day, in the out-patient waiting hall, recently renovated after evacuation by the military, and now for the festive season decorated with almond blossom and choice pink shades enclosing the gigantic electric pendants, an amusing entertainment was given by some of the doctors and medical students.

On Saturday, the 27th, teas and concerts were arranged in all the wards. Viscount and Viscountess Hambleden, together with the Treasurer and other members of the Committee of Management, the honorary physicians and surgeons and their wives, as well as many other friends of the hospital, graced the various wards with their presence. Each nurse was also allowed to invite two friends to tea in the wards that day; many of such friends assisted in entertaining the patients, who again had a right royal time.

On Monday the children had their special party in Short's Memorial Ward, when Father Christmas distributed the gifts with which the great Christmas-tree was laden. The children thoroughly enjoyed themselves, and when evening came there was full evidence that they would one and all enjoy a good night's sleep after the day's excitement.

This brings us to Tuesday, when the nurses had arranged for their enjoyment a fancy-dress party, and there is no doubt as to whether they did enjoy them-

**Christmas in the Hospitals**—(*continued*).

selves. Fancy dress, feasting, and drollery vied with each other for the first place in the memory of those who took part in a memorable affair.

It is specially pleasing to be able to record the fact that through the generosity of subscribers and friends, in response to the appeal made by the Secretary and sister-matron, the whole of the expenses of the festive season were met without encroaching upon the general funds.

So with deep gratitude for all it has been possible to do at King's College Hospital during Christmastide, those responsible for carrying on the gigantic work of this great South London Hospital hope that additional funds will be forthcoming early in the New Year to enable them to wipe out the big deficit and meet the additional expenditure for efficiently maintaining the extra 200 beds which are being placed at disposal for the benefit of the civil patients. To wipe out the debt £50,000 is required, and £60,000 a year more income to meet the above-mentioned increased expenditure.

### ST. GEORGE'S HOSPITAL.

The festivities at St. George's Hospital this year were, as far as possible, similar to those in pre-war years. On Christmas Day there was roast beef and plum-pudding for those patients able to partake of this real old English dinner, and in the afternoon the lady visitors arranged a special tea for the patients, and were present to serve it. Cakes and similar dainties, with crackers and a gift for every patient, were distributed by the visitors in the various wards. There was a Christmas-tree in the children's ward, with a gift for each child in the hospital.

On the 27th a special children's party was given in the out-patient department for children who have been attending in that department, and cakes, buns, and fruit were distributed. In addition, each child received a Christmas gift, and after the tea an entertainment was given for the children, which had been arranged by the nurses especially for the little ones. The children invited to this party were some of the poorest, and in many cases is practically the only festivity they have. The nurses and domestic staff had their dinner of turkey and plum-pudding on Christmas Day. On Christmas Day and the following Sunday the choir-boys from the hospital chapel sang carols in the wards.

The fund for providing the gifts, decorations, and dainties was very kindly contributed by residents in the neighbourhood of the hospital and the lady visitors, so that no part of the cost of the festivities falls on the general funds of the hospital.

### ST. MARY'S HOSPITAL, PADDINGTON.

There were two celebrations of the Holy Communion for the nursing staff, at 6 a.m. and 8.30, both well attended. After the morning service, carols were sung in the hall. The solos in "Good King Wenceslas" were taken by Nurse Brotherton and Nurse Flick, Nurse Wolfe accompanying.

The patients' dinner consisted of turkey and plum-pudding, followed by a liberal supply of apples and oranges. The wards looked very bright. Scarlet was the favourite colour, and there was plenty of it. Alexandra looked very nice in old gold and green. Joy bells of both colours were the ward badge and were in great demand. Crawshay (the children's surgical ward) was in blue, and De Hirsch (children's medical) in pink. The ward teas started at four o'clock. Each patient was allowed one visitor. There were four troupes of residents and students, men and women—"Beauty and the Beast," "Lights Out," "Pierrots," "Mechanical Jane." The little side jokes were both clever and amusing, and brought forth peals of laughter from nurses and patients. Several of the nurses played and sang. Nurse Duncan's singing was greatly appreciated.

### GREAT NORTHERN CENTRAL HOSPITAL.

The arrangements for Christmas at this hospital included his usual gift of Christmas-trees from Lord Islington. Major-General Sir Newton Moore, M.P., and Lady Moore and Lieut. Baldwin Raper, M.P., entertained patients in certain wards. On Christmas Day a special dinner was provided; every patient received a present and was allowed to invite a friend to afternoon tea. A programme of songs and instrumental music took place in the wards. On Boxing Day the members of the Ladies' Association provided Christmas dainties, and every patient received another gift. Carols were sung by a church choir. On Saturday, the 27th, schoolchildren entertained the women's wards with songs and recitations, and Mrs. King Hall, in the ex-soldiers' ward, gave the men under treatment high tea and amused them with a short play.

At the "Summerlee" Hospital of Recovery, East Finchley (a branch of the G.N.C. Hospital), the festive arrangements were greatly helped by the generosity of Mr. Kennedy Jones, M.P. Special entertainment and fare were provided by him, and the Christmas dinner consisted of turkey and plum-pudding. In the evening the patients received a useful present, and were entertained at a concert arranged and carried out entirely by the nursing staff. On Boxing Day a high tea was given at 3 p.m., and on Saturday a concert (arranged by a former patient). At the Reckitt Convalescent Home, Clacton-on-Sea (another branch of the Great Northern Central Hospital), the local residents assisted in the entertainment of the patients.

### KING EDWARD VII. HOSPITAL, CARDIFF.

Christmas 1919 was probably the brightest and happiest Christmas at King Edward VII. Hospital since pre-war days. Certainly the wards never looked more attractive. The discipline of lighting restrictions would seem to have taught the Sisters to apply their decorations without obscuring the light, natural or artificial. Some of the flower schemes were beautiful, and it was really impossible, except by touch, to tell whether many of the flowers were real or artificial.

The festivities started on Christmas Eve, and lasted for about a week. On the afternoon of Christmas Eve the Christmas-tree was illuminated in the Children's Ward, and the gifts distributed. One little boy was wonderfully excited awaiting the advent of Father Christmas. He expected him down the chimney, and kept a sharp look-out for all possible entrances; he had indeed a "priceless time." The Christmas-tree was followed by a really beautiful orchestral and vocal concert in another ward, and later the singers went round to see the other wards, giving little concerts in each.

A feature of Christmas Day was the visit of the Lord Mayor (Councillor Forsdike). Unfortunately, the Lady Mayoress was not well enough to be present, but Miss Forsdike came with her brother, and they spent a most interesting time in the wards. Everyone was glad to see them, and they were glad to see everyone. The Lord Mayor had a kind word for each patient, and it was obvious that he was deeply moved by what he saw. In one small ward, where his greeting to the patients was

**Christmas in the Hospitals**—(*continued*).

particularly intimate (they were all so close together) the men spoke so naturally and unaffectedly about their love and gratitude for the hospital that his Lordship appealed to them, each when he went out into the world again to be a missionary to help the hospital. "Ah!" said one poor man, "I could not describe the kindness and care that has been shown me here."

His Lordship finished up with a visit to the Maternity Hospital which is associated with King Edward's Hospital, where he saw twenty-three new babies, including three pairs of twins. Later in the day the patients immensely enjoyed their Christmas dinner, especially as the turkeys were carved by the various members of the honorary staff, each in the ward with which he is especially identified.

The nurses had their Christmas dinner in "Thomas Andrew's" Ward, which has just been handed over by the builders, and was fortunately empty; otherwise the nurses could not have had their Christmas dinner all together, as the extending hospital has quite outgrown the dining-room accommodation, and Christmas has emphasised the urgency for the provision of a Nurses' Home.

### LIVERPOOL ROYAL INFIRMARY.

Christmas festivities were a great success, and no efforts were spared in giving a time of much happiness to the patients and the staff.

On Christmas Eve a procession of nurses carrying coloured lanterns sang well-known carols through the dimly lighted wards and corridors. The effect was very attractive, and how greatly the patients appreciated it. Christmas Day commenced with each patient, when they opened their eyes, finding on their locker an appropriate gift—according to their circumstances. Great care and thought is always taken by Matron in the choosing of these articles; the military patients were given a pigskin cigarette case filled with cigarettes, or a pipe with tobacco, together with an additional useful gift.

A large number of nurses and patients availed themselves of the privilege of early Communion at 6 A.M. in the prettily decorated chapel; at the service at 10 A.M. the President—Alderman Crosthwaite—read the lessons, and the anthem sung by the choir was "For God so loved the World." Later in the morning Matron, and members of the honorary staff and committee, went round the wards wishing everyone a happy Christmas. At twelve the ward dinners were served. All were fortunate in having turkeys and plum pudding provided, the carving being done by members of the honorary and resident staffs. The ward tables looked most attractive, and were simply laden with various good things. Teas went on during the afternoon, and each ward was an "open house."

At 4.30 the concert party commenced its tour of the wards, those taking part being nurses dressed in prim old English costumes, and the resident staff (including Mr. Rutter our new Secretary Superintendent) in weird and wonderful fancy dress. The latter were extremely popular, and caused much amusement and laughter. It is almost needless to add that this was a very successful feature, and a happy ending to a most enjoyable Christmas Day.

MILITARY WARD. LIVERPOOL ROYAL INFIRMARY. CHRISTMAS 1919.

The Decorations.

The ward decorations were charming, each sister having shown considerable individuality in evolving her own scheme, the lampshades being again particularly distinctive. The large soldiers' ward was very original; on entering one was attracted by a number of owls of various sizes peering in all directions from huge bare branches; yellow being the prevailing colour of the shades, etc., formed an artistic background to the sombre black, white, and grey of the owls, the whole of which had afforded much pleasure in the making by the soldiers for many days.

The large central hall in the Nurses' Home was the scene of a very realistic snowstorm under which the nurses ate their Christmas dinner, the whole staff at the same time, while the sisters took care of the wards, the "cold touch" being taken away by holly and the pink and yellow shades. The sisters had their Christmas dinner amidst the raging of the same snowstorm the following evening.

Saturday, December 27, the nursing staff was "At Home" to their personal friends. Tea was provided in the Home, and the wards visited afterwards.

At the nurses' fancy dress party on Tuesday, December 30, the popular dance is to be the order of "the night," and prizes given by ballot for the most original, ingenious, and attractive characters.

The whole of the expenses of the festivities have been defrayed out of a special Christmas fund, to which generous contributions were made by the many friends of the Hospital.

**[We regret we are compelled to hold over till next week accounts from many other hospitals.—Editor "The Hospital."]**

## Figures and Tables

**Figure f1:**